# Do stereotypies help or harm? Exploring the link between cortisol level and abnormal behaviours in animals: a review

**DOI:** 10.1186/s12983-025-00576-0

**Published:** 2025-08-13

**Authors:** Weronika Helena Hildebrand, Grzegorz Zaleśny

**Affiliations:** Institute of Biology, Wrocław University of Environmental and Life Sciences, 5B Kożuchowska Street, 51-631 Wrocław, Poland

**Keywords:** Chronic stress, Stress response, Stereotypical behaviour, Abnormal behaviour, Cortisol, Zoo animals, Horses, Dogs

## Abstract

Stereotypical behaviours in animals, often linked to stress, are repetitive actions that may lack a clear purpose. This review examines the relationship between cortisol levels, a key stress hormone, and stereotypic behaviour across various animal species, including zoo mammals, horses, and dogs. A total of 99 studies were analysed to understand whether elevated cortisol levels are associated with stereotypies. While many studies report a positive correlation between cortisol and stereotypical behaviour, more detailed research suggests that these behaviours may also serve as coping mechanisms, potentially reducing cortisol levels. The findings highlight the complexity of this relationship, as some studies show a negative or no correlation at all. To address these inconsistencies, this review critically examines the existing evidence and aims to provide a clearer understanding of the relationship. The review emphasises the need for further research, including long-term studies and the inclusion of additional hormonal markers, to clarify whether stereotypical behaviours only reflect chronic stress or also represent stress-relieving adaptive responses.

**Supplementary Information:**

The online version contains supplementary material available at 10.1186/s12983-025-00576-0.

## Introduction

Stress is an inevitable part of life for all animals. Free-living animals face various stressors, including the need to survive, hunt for food, and care for their offspring. In contrast, animals kept in captivity often experience different stress factors that arise primarily from their inability to engage in natural behaviours, limited space, or lack of environmental enrichment. These stressors can vary significantly depending on species, individual temperament, and living conditions. The specific stressors animals encounter can also vary significantly depending on the species and their living conditions. When such stress becomes chronic or overwhelming, it can lead to a wide array of behavioural disturbances, including the development of stereotypical behaviours—also known as stereotypies [1, 2].

Stereotypic behaviour refers to repetitive actions that seem to lack a specific purpose. These behaviours often arise from frustration, repeated attempts to cope, or issues related to brain function (e.g., environmental deprivation, neurodevelopmental disorders, neurological injury). Generally, these behaviours result from the stress an individual is predisposed to experience. This can include typical and harmless behaviours, such as a cat kneading a pillow, as well as abnormal behaviours that might indicate an underlying issue. For example, hand-flapping in individuals with autism may reflect a neurological or developmental condition. It can serve either as a symptom of the condition or as a coping mechanism in response to environmental stress [1–3]. It's important to note, however, that beyond specific stereotypies, the literature also describes a broader spectrum of stress-related behaviours that constitute a form of abnormal behaviour and reflect compromised welfare, even if they don't meet the strict criteria for stereotypy. These may include, for instance, avoidance, hypervigilance, withdrawal, or exaggerated startle responses [3–6].

Within this category, “stereotypies” refer to more rigid and repetitive behaviours linked to brain dysfunction. The broader term encompasses behaviours involving a variety of movements that don’t appear to serve a clear function [3]. Stereotypic behaviour is observed in all animals and humans, varying according to the conditions in which the animals live, their species, and other factors. While stereotypies, such as pacing or excessive grooming, are often the focus of studies, it is equally important to consider additional behavioural indicators to fully assess an animal’s response to stress [3–6].

According to research [3], the prevalence of stereotypies can be as high as 100% in certain animal groups, particularly in farm and laboratory animals, where many express some form of abnormal behaviour at least once in their lives. Another group frequently exhibiting stereotypy—and thus the subject of extensive study—includes animals in facilities such as zoological gardens, breeding centres, and rescue organisations. Although efforts have been made recently to improve the overall welfare of animals in zoological gardens, current literature indicates that stereotypic behaviours still affect a significant percentage of their populations [3–6]. This suggests that even with improvements in husbandry, environmental design, and enrichment protocols, some animals continue to experience stress or long-term behavioural effects due to earlier deprivation or ongoing unmet needs.

Finally, stereotypies are also observed in horses and domestic animals, such as cats and dogs that live in close proximity to humans. Various studies aim to determine the factors that predispose horses to behaviours like crib-biting, head bobbing, and tail pawing, as well as to find solutions to prevent or stop these disruptive behaviours [1, 3–6]. In companion animals, stress-related behaviours are often linked to confinement, social isolation, or training methods, making them important targets for welfare interventions [3–6].

In addition to observable behavioural changes, there are various methods for assessing the level of stress in animals. One commonly used method involves measuring cortisol and its metabolites. Cortisol is a hormone produced by the adrenal glands in response to stress, playing a crucial role in the body's “fight or flight” response. This hormone is primarily released in reaction to acute stressors through the hypothalamic–pituitary–adrenal (HPA) axis. Cortisol helps mobilise energy by increasing blood sugar and blood pressure while promoting analgesia (pain relief) [7, 8]. At the same time, it conserves energy by suppressing non-vital functions, such as reproduction, immune responses, and digestion. While essential for short-term survival, prolonged exposure to cortisol can lead to adverse health effects due to the wear and tear it causes on the body [8]. Cortisol levels can be easily monitored in both domestic and wild animals. It can be measured through various biological samples, including blood, saliva, hair, urine, and faeces [8–10]. The choice of biological matrix influences how well cortisol reflects both acute and chronic stress, making it crucial to align measurement methods with study goals.

The question that remains is whether there is a correlation between cortisol levels in animals and the occurrence of abnormal or stereotypic behaviour. In this study, we present a review of 99 articles that investigate cortisol levels and their relationship with behaviour in zoos and captive mammals, horses, and dogs. We did not include studies focusing on laboratory or farm animals, as these populations are often housed under standardised conditions specifically designed to minimise stress and behavioural variability for experimental control or productivity [3]. In contrast, our focus was on animals in more variable and enrichment-sensitive environments, where the relationship between cortisol and behaviour may be more reflective of welfare challenges relevant to companion and zoo animals. Despite the volume of research in this field, findings remain inconsistent across species and study designs. Some studies report strong correlations between cortisol levels and behavioural indicators of stress, while others find little to no association. By examining this diverse body of research, we aim to clarify existing patterns, identify methodological limitations, and contribute to a more nuanced understanding of how physiological and behavioural data can inform animal welfare assessment.

## Materials and Methods

A comprehensive literature search was conducted using the Scopus database to identify studies containing the terms “stress”, “repetitive”, “stereotypy”, “stereotyped”, “stereotypies”, “stereotypic”, or “stereotypical” in conjunction with “cortisol”, “corticoid”, or “glucocorticoid”. The search was further refined to target studies focused on specific groups: “zoo animals”, “captive animals”, “mammals”, “horses”, “equine”, “equids”, “canine”, “canids”, or “dogs”.

The initial search yielded 1048 records. Only original research articles were included; review articles were excluded, resulting in 877 eligible studies. Further refinement excluded studies conducted on farmed or laboratory animals, narrowing the dataset to 780 articles. To maintain focus on mammalian species, studies involving birds, fish, reptiles, and other non-mammalian groups were excluded, resulting in 620 remaining articles.

Each of these 620 records was then manually reviewed by the author to ensure the inclusion of only the most relevant studies and to provide a comprehensive and accurate review. This manual verification involved reading the titles, abstracts, and, when necessary, the full texts to assess the relevance and quality of each study based on predefined inclusion criteria, such as the species studied, cortisol measurement methods, and focus on stereotypical behaviours. Studies that lacked sufficient methodological detail or did not directly address the research question were excluded from the analysis. This step was conducted carefully to avoid errors and ensure the highest standards of data reliability.

For each study, information was extracted regarding the animal species investigated, the materials and methods used for cortisol measurement, and the findings concerning cortisol levels and abnormal or stereotypic behaviours. Studies lacking specific information on either cortisol measures or stereotypic or stress-related behaviours were excluded from the final selection.

As a result, we included 70 articles published between 1993 and 2024 that focused on zoo and captive mammals, 21 articles published between 1998 and 2024 on horses, and eight articles published between 2005 and 2022 on dogs.

## Results

### The method of measuring cortisol or its metabolites

The most frequently used material for measuring cortisol and its metabolites was faecal samples, with a total of 48 studies utilising them. The second most common material was blood (serum/plasma) samples for cortisol measurements. Other materials included urine, hair, and saliva. Only eight studies used more than one type of material for cortisol measurements. Detailed data are presented in Table 1.

**Table 1:** Representation of the number of studies that used each type of biological material for the measurement of cortisol or its metabolites

Material used/no of studies	Zoo animals	Equine	Dogs	Total
Urine	9	0	1	10
Faeces	43	2	3	48
Blood (serum/plasma)	8	17	2	27
Hair	7	1	0	8
Saliva	6	5	2	13
More than one of above	5	4	0	9

### Methods used for cortisol measurements

The two most commonly used methods in the reviewed studies are radioimmunoassay (RIA) and enzyme-linked immunosorbent assay (ELISA), also known as enzyme immunoassay (EIA). RIA was selected as the method in 32 studies, while EIA was used in 61 studies. Additionally, seven studies included other methods, such as High-Performance Liquid Chromatography (HPLC) or Electrochemiluminescence (ECL) [11–13]. Four studies did not mention a specific method. Only one study employed more than one method for cortisol measurements [13]. The methods used, categorised by three animal groups, are presented in Table 2.

**Table 2:** Representation of the number of studies employing each laboratory method for the measurement of cortisol or its metabolites

Method used/no of studies	Zoo animals	Equine	Dogs	Total
EIA	45	13	3	61
RIA	20	8	4	32
Other/unknown	5	0	1	6

### Species of animals included in the studies: zoo mammals

Various studies have examined a diverse range of over 44 species across multiple taxonomic groups. Primates and Carnivora were the most represented, with 18 species in the first group and 17 in the second. Primate species included great apes such as *Pan troglodytes* and *Pongo pygmaeus* [14–17]*,* as well as Old and New World monkeys like *Macaca fascicularis* and *Cebus apella* [12, 18–29]. Carnivores included species such as large felids (*Panthera tigris*, *Acinonyx jubatus*) [30–35, 43], bears (*Ailuropoda melanoleuca*, *Ursus maritimus*) [13, 36–40], and smaller carnivores (*Cryptoprocta ferox*) [41]. Artiodactyls were represented by six species, such as *Giraffa camelopardalis* and *Camelus dromedarius* [42–45], while two species of Perissodactyls, primarily elephants (*Elephas maximus*, *Loxodonta africana*), were included [46–51]. Studies on marine mammals featured one cetacean—*Tursiops truncatus* [52]*,* and a single marsupial—*Lasiorhinus latifrons* [53]*.* The genera most often studied were *Maccaca* sp. (11 studies) [12, 18–25, 27, 29] and *Ailuropoda* sp. (5 studies) [13, 36, 37, 39, 40]. Two of the articles included three or more species of animals [54, 55]. The most commonly studied orders were Carnivora and Primates. All species are segregated into the orders to which they belong, and the number of studies examining them is presented in Table 3.

**Table 3:** Representation of animal orders, families, and species included in the reviewed studies, along with the number of studies and corresponding references

Order	Family	Species	No of studies/Species	References	No of studies/Order
Artiodactyla (Even-toed ungulates)	Bovidae	*Antelope cervicapra*	1	[56]	8
	Camelidae	*Camelus dromedarius*	2	[42, 45]	
	Cervidae	*Axis porcinus*	1	[57]	
	Giraffidae	*Giraffa camelopardalis*	2	[43, 44]	
	Moschidae	*Moschus chrysogaster*	1	[58]	
		*Moschus berezovskii*	1	[59]	
Carnivora (Carnivores)	Canidae	*Canis rufus*	1	[60]	26
	Felidae	*Acinonyx jubatus*	2	[30, 33, 43]	
		*Caracal caracal*	1	[54]	
		*Felis bengalensis*	1	[61]	
		*Leptailurus serval*	3	[54, 62, 63]	
		*Leopardus pardalis*	1	[54]	
		*Neofelis nebulosa*	2	[64, 65]	
		*Panthera leo*	1	[31]	
		*Panthera onca*	1	[66]	
		*Panthera pardus*	3	[35, 63, 67]	
		*Panthera tigris*	4	[31, 32, 34, 35]	
		*Uncia uncia*	2	[63, 68]	
	Eupleridae	*Cryptoprocta ferox*	1	[41]	
	Ursidae	*Ailuropoda melanoleuca*	5	[13, 36, 37, 39, 40]	
		*Helarctos malayanus*	1	[69]	
		*Melursus ursinus*	1	[70]	
		*Ursus maritimus*	2	[38, 65]	
Cetacea (Marine Mammals)	Delphinidae	*Tursiops truncatus*	1	[52]	1
Perissodactyla (Odd-toed ungulates)	Elephantidae	*Elephas maximus*	3	[46, 49, 50]	6
		*Loxodonta africana*	3	[47, 48, 51]	
Primates (Primates)	Atelidae	*Alouatta caraya*	1	[55]	28
	Callitrichidae	*Callithrix jacchus*	2	[71, 72]	
		*Saguinus oedipus*	1	[73]	
	Cebidae	*Cebus apella*	2	[26, 28]	
		*Sapajus libidinosus*	1	[74]	
	Cercopithecidae	*Macaca spp.*	11	[12, 18–25, 27, 29]	
		*Papio papio*	1	[75]	
	Hominidae	*Gorilla gorilla gorilla*	2	[15, 17]	
		*Pan paniscus*	1	[76]	
		*Pan troglodytes*	2	[16, 17]	
		*Pongo pygmaeus*	2	[14, 55]	
	Hylobatidae	*Hylobates lar*	1	[55]	
		*Symphalangus syndactylus*	1	[55]	
	Lemuridae	*Hapalemur alaotrensis*	1	[77]	
		*Lemur catta*	1	[78]	
		*Varecia rubra*	1	[55]	
	Rhinopithecidae	*Rhinopithecus roxellana*	1	[79]	
	Aotidae	*Aotus spp.*	1	[80]	
Diprotodontia (Marsupials)	Vombatidae	*Lasiorhinus latifrons*	1	[53]	1

The methods used to measure glucocorticoids varied among the studies reviewed. While some studies measured cortisol directly, typically through plasma or saliva samples, others assessed faecal glucocorticoid metabolites (FGM), which are breakdown products of cortisol excreted in the faeces. Although FGM does not measure circulating cortisol levels directly, it is widely accepted as a reliable, non-invasive indicator of hypothalamic–pituitary–adrenal (HPA) axis activity over time [7–10]. Given that both direct cortisol measurements and FGM reflect glucocorticoid activity associated with stress responses, and to maintain clarity and consistency throughout the text, the term “cortisol” will be used to refer to both direct and metabolite-based assessments unless a distinction is necessary for interpretation.

### Cortisol level and behaviour relationship in zoo and captive mammals

#### Positive cortisol–abnormal or stereotypical behaviour relationship

Twenty-nine of the studies reviewed revealed a generally positive relationship between cortisol levels and abnormal behaviour, typically in terms of the frequency and/or duration of such behaviours. In some cases, the studies also considered the intensity or specific forms of abnormal behaviour, such as pacing, self-directed actions, or vocalisations, in relation to cortisol concentrations. However, only a subset of these studies specifically examined a direct correlation—that is, they quantitatively assessed whether increases in stereotypical behaviour were statistically associated with elevated cortisol levels within the same individuals or groups, using concurrent behavioural observations and physiological measurements [12, 26, 35, 37, 38, 44, 49, 50, 58, 61, 69, 70, 72, 75, 76, 81].

Pomerantz et al. [26] investigated the relationship between two types of stereotypical behaviour and cortisol levels in primates, as well as their corresponding emotional states. They found a positive correlation between the rates of head twirls. However, no correlation was found between cortisol levels and the time spent engaging in pacing. In a separate study, Shepherdson et al. [38] also reported a positive correlation between pacing and cortisol levels in polar bears (*Ursus maritimus*). Brand et al. [76] investigated self-directed stereotypy in primates and noted a positive correlation between cortisol and hair-plucking behaviour in female bonobos (*Pan paniscus*). This correlation was not observed in male bonobos. The authors suggested that further research is needed and indicated that hair-plucking might be a stress-induced behaviour limited to females in this species. Another example of a positive correlation between cortisol levels and the presence of stereotypical behaviour was presented by Zhou et al. [58]. Their study on Alpine musk deer (*Moschus chrysogaster*) found that animals exhibiting stereotypical behaviour had significantly higher faecal cortisol metabolite levels than animals not exhibiting such behaviour. Additionally, Lv et al. [81] conducted another study on musk deer (*Moschus chrysogaster*) that found higher cortisol levels in a high-density housing group compared to a lower-density group. This study also indicated that animals in high-density conditions displayed longer and more frequently occurring stereotypical behaviours, such as eating foreign materials, wall jumping, and others.

Some studies incorporated external factors, such as the addition of enrichment to the animals'environment [12, 15, 37, 54, 61, 69, 70, 75]. Abdul-Mawah et al. [69] compared semi-captive bears with fully captive individuals housed in three separate facilities. They found that the semi-captive bears had significantly lower cortisol levels and exhibited less stereotypical behaviour than their fully captive counterparts. Furthermore, the introduction of environmental enrichment led to a decrease in cortisol levels across all captive bears, suggesting a positive impact of enrichment on stress reduction. A similar effect was observed in the study of Radical et al. [54], who observed an increase in affiliative behaviour among felids following the introduction of enrichment. The most significant difference in cortisol levels was found between ocelots before and after enrichment. Umsook et al. [70] described differences in cortisol levels and the presence of stereotypical behaviour before, during (only for stereotypies), and after the enrichment periods. A direct correlation was also found between stereotypical behaviour and cortisol levels. In addition to enrichment, Carlstead et al. [61] included housing conditions in their study. The findings indicated that stress behaviours increased when the cats were housed in barren cages. Concurrently, the study monitored urinary cortisol levels, finding that these levels were elevated in the barren housing conditions and decreased when the cats were provided with environmental enrichment and hiding places. However, the authors noted that cortisol levels do not always correlate with stereotypical pacing behaviours, even though they did find a correlation with some abnormal or stress-related behaviours in their study. Housing conditions were examined in the studies conducted by Koyama et al. [12]. The authors found that paired housing significantly reduced the incidence of stereotypical behaviours and resulted in decreased cortisol levels. Another type of enrichment was investigated by Fagot et al. [75], who utilised a computerised learning system to assess its effects on primates. Their study demonstrated that providing access to these computerised testing systems resulted in fewer resting periods and a reduction in stereotypical behaviours among the animals.

Another study investigating the effects of enrichment focused on the stereotypical behaviour of giant pandas (*Ailuropoda melanoleuca*) [37]. This study, in contrast to previous findings in other species, did not find a negative correlation between the presence of enrichment, stereotypical behaviour, and cortisol levels. However, the authors did identify a relationship between the level of stereotypy displayed by the pandas and their cortisol concentrations. Notably, this correlation was observed during the oestrus phase of the reproductive cycle. Additionally, the study established a connection between behavioural changes and the different stages of the reproductive cycle in giant pandas, suggesting that reproductive status may influence both cortisol levels and stereotypic behaviour [37].

Kaplan et al. [72] researched the differences between indoor and outdoor environments for common marmosets (*Callithrix jacchus*). They found that outdoor enclosures were associated with less stress-related behaviour, such as auto-grooming and scent marking. This decrease in stress behaviours correlated with lower cortisol levels. Elevated cortisol levels were observed during stressful situations, such as roof repairs and hailstorms. The study also revealed different correlations related to the types of calls made by the primates; there was a negative correlation for alarming “tsik” calls and a positive correlation for contact “phee” calls.

Interestingly, some studies have identified a positive cortisol-stereotypical behaviour relationship only in some of the individuals studied [15, 50]. Schmid et al. [50] measured cortisol levels of elephants (*Elphas* sp.) and noted a positive correlation between cortisol levels and stereotypical behaviour in one individual. Similarly, Leeds et al. [15] reported a correlation in only one individual. In their study, Vaz et al. [35] compared the stereotypical behaviour of two species of felids and their cortisol levels. They observed Royal Bengal tigers (*Panthera tigris tigris*) to be more engaged in stereotypical behaviour (83% engaged in stereotypies) than Indian leopards (*Panthera pardus fusca*) (62%). The researchers examined various internal and external factors, including age, sex, enclosure size, the presence of different types of habitat enrichment, and the attitudes of caretakers, to establish a positive relationship between cortisol levels and stereotypical behaviour. However, the authors emphasise that species differences must be taken into account when improving animal living conditions. They provide evidence that certain enrichment strategies led to decreased cortisol levels in one animal group, but not in another. For example, increasing enclosure size improved both behaviour and cortisol levels in tigers, but did not have the same effect on leopards. Jain et al. [44] investigated abnormal behaviour in giraffes (*Giraffa* spp.) and measured the impact of an external stressor on their faecal glucocorticoid metabolites. They observed both behavioural and cortisol changes in one individual after the death of their companion. During the construction period, behavioural changes were observed in individuals. At the onset of the stressor, both giraffes exhibited elevated cortisol levels. In one giraffe, cortisol levels decreased over time, while in the second individual, they remained elevated.

Some authors do not directly examine stereotypical behaviours but observe a positive relationship between certain types of abnormal behaviour and cortisol levels. Zhang et al. [79] researched behavioural changes and cortisol level fluctuations in primates after group division. They observed a decrease in cortisol levels alongside a reduction in aggressive behaviour. Additionally, cortisol levels increased following a stressful relocation period. Relocation stress has also been researched by Dettmer et al. [22]. They found a correlation between hair cortisol levels and anxiety-related behaviour in primates after the relocation. Another study, including the period of relocation by Laws et al. [49], highlighted a significant rise in faecal cortisol metabolite levels (389% and 340%) following relocation, with peak levels occurring two days after the move. Elevated cortisol levels persisted during the establishment of the new herd. After relocation, an elephant studied showed a 400% increase in stereotypic behaviours, and the authors also reported disruptions in sleep patterns.

Some of the studies included an external stressor, such as human contact, in animals. Hogan et al. [53] researched the effect of human contact on wombats in captivity. The study found that regular handling altered the human–wombat relationship by reducing the animals'reactivity and avoidance behaviours toward human handlers. However, despite these behavioural changes, the study observed that the stress response, as indicated by faecal cortisol metabolite levels, was not reduced by regular handling. This suggests that while wombats may become less reactive to human presence over time, their physiological stress response remains unchanged. The cortisol levels after handling were significantly higher than before. The effect of human handling and contact was also researched by Akbar and Evans [18]. Their study included research on the so-called “dancing monkeys” used for entertainment in Pakistan. The study confirmed that “dancing monkeys” had significantly higher cortisol levels than the control group. Another study examining the impact of humans on the stress levels of animals was conducted by Rajagopal et al. [56].

Moreover, a significant difference was observed between days with high and low visitor density. The changes have been seen in animals’ behaviour as well as in their faecal cortisol levels. Similar changes were observed by Fink et al. [43], who conducted a study to investigate the effect of prolonged zoo closures during the COVID-19 pandemic. They, however, noted significantly lower changes and suggested that those might have been a result of other procedures and stressors rather than the presence of the visitors. Higher cortisol levels were observed during the transition periods (closure/re-opening of the facility only); similar changes were observed for behavioural stress responses. Visual contact with human visitors was researched by Sherwen et al. [28]. In their research, a reduction in aggressive behaviour and other abnormal behaviours was noted when visitors were absent. The primates were also seen avoiding human contact. Significant changes in faecal cortisol metabolite levels were observed between the periods of visitor presence and absence.

The effect of cortisol on reproductive function was researched by Fontani et al. [73]. Besides behavioural changes and cortisol levels, reproductive hormone levels in males were also included in the study. Correlations have been observed between cortisol levels and aggressive behaviours, as well as between cortisol levels and affiliative behaviour, in two facilities. The effect of the mating season was also researched by Marliani et al. [41]. The authors noted higher cortisol levels in animals during the outdoor mating season. During that time, females presented with a shorter duration of behaviours such as locomotion, marking, and seeking, but males were observed with the abnormal behaviour of pacing. Testosterone, along with cortisol, was measured by DeCaluwe et al. [64] in their research. Animals described as “anxious” had higher cortisol peaks than the animals described as “calm”.

One study [52] explored the relationship between cortisol levels and what they refer to as “higher behavioural diversity”. Their findings suggest that animals with elevated cortisol levels tend to be less active and show fewer behavioural changes over time compared to those with lower cortisol levels. This observation might indicate stress or anxiety in these animals.

#### Negative cortisol–abnormal or stereotypical behaviour relationship

Conversely, some studies present conflicting results and suggest entirely different conclusions. Six studies [27, 46, 48, 63, 65, 71] identified a negative relationship between cortisol levels in animals and the presence of stereotypical or abnormal behaviour. These studies suggest that the exhibited abnormal or repetitive behaviours, often arising due to high stress, may serve as a coping mechanism, helping to reduce the stress levels within the organism [48, 65]. Shepherdson et al. [65] in their study found that, in some instances, spending more time engaging in abnormal or stereotypic behaviours was linked to lower concentrations of faecal glucocorticoid metabolites (FGM). While there were no significant differences in mean cortisol levels, the maximum cortisol values revealed notable differences in cortisol levels. Bears that did not display stereotypic behaviour exhibited higher peak cortisol levels and greater variability in their cortisol levels compared to those that did show such behaviours. This suggests that non-stereotypic polar bears may be more responsive to environmental stimuli or perceive their environment as more stressful. A negative correlation was observed in one of the elephants studied by Kelling et al. [48]. Notably, stereotypical swaying was significantly associated with decreased cortisol levels in this individual, suggesting that such behaviour may serve to reduce arousal. The researchers measured baseline cortisol levels after the elephant had swayed for some time. In one case, cortisol decreased after 30 min of engaging in this stereotypical behaviour. However, the difference was not statistically significant.

In a study by Chosy et al. [63], researchers investigated the impact of an external factor, specifically, construction sites as a stressor for felid species. They collected faecal samples and conducted behavioural observations on five individuals from four different felid species before, during, and after the construction period. The results revealed that, on average, the z-score for faecal glucocorticoid metabolite concentration increased during construction compared to baseline levels. While these elevated levels persisted after construction ended, they showed a tendency to return to baseline levels. Furthermore, all individuals exhibited a significant reduction in both the frequency of pacing and the time spent visible during the construction phase.

Bansiddhi et al. [46] also considered various external factors, including work routines, walking, restraint, rest areas, and foraging opportunities. Another study by Pomerantz et al. [27] assessed the rise in cortisol levels among primates, comparing individuals with stereotypical and non-stereotypical behaviours. The findings indicated that those exhibiting high levels of self-directed stereotypy experienced less pronounced increases in cortisol following an acute stress challenge than those with lower rates of stereotypy.

Additionally, one study [33] identified a negative correlation between behavioural diversity and cortisol levels, which stands in contrast to the findings of the study. In addition, there is a significant inverse relationship between behavioural diversity and the concentrations of faecal glucocorticoid metabolites. In a separate experiment involving primates [71], researchers measured cortisol levels across various groups and initially assessed them in relation to stress-related behaviours. They discovered a negative correlation between cortisol levels and behaviours such as auto-grooming and scratching. In contrast, a positive correlation was found between cortisol levels and behaviours including locomotion, individual piloerection, and scent marking, which may suggest signs of anxiety.

Finally, two studies noted a possible negative correlation but suggested that further studies are necessary. Albanese et al. [19] measured cortisol levels during and between enrichment sessions. However, higher cortisol levels were observed during the enrichment time, when the frequency and duration of stereotypical behaviour decreased. The authors suggest a possible negative correlation but recommend further research. They hypothesise that a longer study could confirm that, despite higher cortisol levels during enrichment sessions, such enrichment might ultimately reduce cortisol levels chronically. The effect of enrichment has also been researched by Zaragoza et al. [17]. They noted an increase in cortisol levels in gorillas after enrichment was introduced. The change has not been seen in chimpanzees. Moreover, both species exhibited more exploratory and manipulative behaviour during enrichment sessions, and the repetitive behaviour has also been reduced. This might suggest a possible negative correlation between cortisol levels and abnormal behaviour.

### No correlation between cortisol level and stereotypical or abnormal behaviour

Sixteen studies on captive mammals found no correlation between cortisol levels and the presence of abnormal or stereotypical behaviour [13, 14, 16, 21, 24, 29–31, 34, 39, 40, 51, 62, 66, 68, 80]. Additionally, in some cases, a slight relationship was noted, but the authors described it as statistically insignificant [20, 77]. One study hypothesised a possible link between cortisol levels and stereotypical behaviour based on its findings; however, no such correlation was found in later phases of the research [31]. According to van Dam [31], the felids studied demonstrated elevated cortisol levels, ranging from 118 to 1225% of baseline levels, when construction activities, such as fence removal, occurred in their close vicinity. Snyder et al. [39] investigated the effect of transportation on stress levels in animals. Although cortisol levels increased during the transportation period, no behavioural changes were observed. Acaralp-Rehnberg et al. [62] investigated the impact of encounter programs on the adrenocortical activity of the *Leptailurus serval*. The researchers observed a decrease in stereotypical activity time, but no correlation was found between this decrease and cortisol levels. No correlation was found between behavioural changes and cortisol levels in African bush elephants (*Loxodonta africana*) [51]. The study found that implementing scheduled activities in elephants may reduce the incidence of stereotypical behaviours, but each animal exhibited distinct cortisol levels. The results and additional information about studies can be found in Supplementary Table 1.

### Unclear relationship or other factors measured

Several studies did not address the direct relationship between various stress factors and cortisol levels in animals. Aubè et al. [42] observed daily fluctuations in abnormal behaviour among animals in their studies. This suggests that these variations may be linked to daily hormonal changes (circadian rhythm), as indicated by research [7, 42]. The authors, however, noted a negative correlation between stereotypical behaviour and cortisol levels that was especially marked before and after food administration to animals. Further investigation into this relationship could provide valuable insights into the factors influencing animal behaviour [42]. In their study [74], researchers measured cortisol levels alongside behaviours that could indicate stress and normative behaviour. They identified differing correlations based on the type of stress-related behaviour examined. A negative correlation was observed for behaviours such as “Ingestion/Self-scratching,” while a positive correlation with cortisol levels was found for other behaviours, including “Stereotyped and Self-Directed”. Pollastri et al. [78] included primates from a walk-in facility where visitors were in close contact with the animals. Cortisol levels were higher during periods of high visitor density than during periods of lower density. Behavioural results showed that the control groups performed more diverse behaviours than during weekends. No direct correlation with stereotypic behaviour is marked within the study [78].

Other studies have measured cortisol levels in animals under various stress situations, including transportation [39], diet changes [60], radio collaring [36], introduction of geofencing devices [47], maternal separation [23], and crowding [59]. While some research confirmed elevated cortisol levels in potentially stressful situations [23, 47, 60], other studies have reported no significant differences in cortisol levels [36]. Interestingly, one study [32] compared cortisol levels in tigers living in captivity, free-ranging facilities, and natural reserves and found no significant relationship, suggesting that factors other than space may influence stress in these animals. However, the same study described a difference in cortisol levels of animals on and off display. In a notable finding, He et al. [59] investigated forest musk deer, typically solitary animals in the wild, to assess the impact of crowding on cortisol levels.

Contrary to expectations, the researchers discovered that female deer in more crowded enclosures exhibited lower faecal cortisol levels, highlighting the complexity of animal behaviour in captivity. Similarly, Majchrzak et al. [45] focused on working camels. They found that these animals had lower cortisol levels following potentially stressful rides, indicating that their stress response may be more nuanced than previously thought.

Additionally, Vaglio et al. [55] assessed the effects of scent enrichment on various primate species, finding diverse outcomes. In some groups, scent enrichment led to increased cortisol levels and affected social interactions negatively, while in others, it resulted in decreased cortisol levels (not statistically significant). Different types of enrichment were also studied by Panchal et al. [67], and the positive effect of bigger enclosures on welfare was confirmed.

Finally, Wang et al. [13] examined the relationship between mating behaviour and cortisol levels, but no correlation was found. The effect of hormonal changes and the menstrual cycle was studied by Kim et al. [25]. What they found was that non-menstruating individuals may be more stressed than the menstruating ones.

The summary of studies on captive mammals is presented in Table 4.

**Table 4:** Summarisation of studies on captive mammals, including type of abnormal behaviour, type of correlation and the reference number

Type of behaviour/stereotypy	Correlation type	Number of studies (total: 70)	References
*Oral and self-directed stereotypies* (overgrooming, self-scratching, hair-plucking, chewing paws, tail-sucking, eating foreign material and other)	Positive	7	[18, 35, 44, 58, 72, 76, 81]
	Negative	3	[19, 27, 71]
	No correlation	9	[14, 20, 21, 24, 29, 30, 39, 66, 77]
	Unclear	3	[42, 55, 74]
*Locomotory stereotypies* (pacing, weaving, head twirls, swaying, rocking and other)	Positive	16	[12, 26, 35, 37, 38, 41, 43, 44, 49, 50, 58, 61, 69, 70, 75, 81]
	Negative	4	[19, 27, 48, 63]
	No correlation	14	[20, 21, 29–31, 34, 39, 40, 51, 62, 66, 68, 77, 80]
	Unclear	6	[36, 42, 47, 55, 59, 74]
Other/not specified	Positive	16	[12, 18, 22, 28, 37, 43, 49, 52–54, 56, 58, 64, 72, 73, 79]
	Negative	6	[17, 19, 27, 33, 65, 71]
	No correlation	11	[13, 14, 16, 20, 21, 31, 34, 39, 40, 66, 77]
	Unclear	10	[23, 25, 32, 36, 45, 55, 57, 60, 74, 78]

### Cortisol level and abnormal/stereotypical behaviour relationship in horses

#### Positive cortisol–abnormal or stereotypical behaviour relationship

Seven studies on horses have explored the relationship between cortisol levels and abnormal and stereotypical behaviour, revealing a generally positive correlation [82–88].

However, it is essential to note that some research calls this correlation into question, as the cortisol dynamics do not consistently demonstrate a clear link with behavioural changes. For example, Bachmann et al. [84] highlight that although stereotypical horses typically have higher cortisol levels, these levels do not rise following exposure to a stressor. This observation may suggest a potential habituation to stress due to chronic exposure.

Furthermore, findings of McGreevy and Nicol [87] indicate that while horses with stereotypical behaviours, such as crib-biting, showed elevated cortisol levels compared to non-stereotypical horses, these levels did not increase when the crib-biting was curtailed. The increase in cortisol level was, however, seen when the crib-biting horses were prevented from eating. The research of Carvalho Seabra et al. [85] also contributes valuable insights, indicating that horses in single-housing conditions exhibited stereotypical behaviour, along with higher cortisol levels. However, the prevalence of abnormal behaviours among almost all the animals studied limited the possibility of establishing a control group for comparison.

It is noteworthy that in certain studies, while the correlation between cortisol levels and behaviour is not explicitly measured or named, it can still be inferred from the behavioural data results and cortisol level measurements presented [86, 88]. Hanis et al. [86] included food changes as a factor influencing animal stress responses and general welfare in their study. Their general observation was that horses exhibiting abnormal behaviour had significantly higher cortisol levels than the control group. Within the experiment, horses presenting abnormal behaviour were fed a high forage, high fibre diet and their behaviour, blood parameters, leptin, and ghrelin concentrations were sought. They noted a 30% decrease in cortisol levels after the induction of the changed diet. The differences were also evident in stereotypic behaviour and redirected behaviours, which decreased by approximately 70% and 86%, respectively. The authors, therefore, suggest that a simple dietary change can significantly improve animal welfare. The aspect of the effect of diet was also included in research by Arena et al. [82]. The authors suggest that a high-fibre and high-energy diet may contribute to the appearance of pathological behaviours, such as stereotypies.

Moreover, the authors compared cortisol levels in horses expressing pathological behaviours and healthy individuals. A significant difference was observed between the groups, with pathological horses exhibiting higher cortisol levels. Moreover, a correlation was identified between pathological behaviour and the duration spent either in the paddock or inside a stable. Horses that spent more time indoors tended to have elevated cortisol levels.

Schmucker et al. [88] examined cortisol levels following group formation and the relocation of horses to individual stable conditions, which may act as a stressor. The initial move did not impact cortisol levels, unlike the subsequent type of relocation. A significant increase in plasma cortisol levels was observed, with levels returning to baseline after eight days. The authors also assessed immune response alongside cortisol measurement in their study. The impact of housing systems was also discussed in research by Baboo et al. [83]. The findings revealed that horses in single housing systems with no interaction and those in pairs exhibited lower cortisol levels compared to horses in single housing with limited contact and those in group settings. It is crucial to note that the horses were transitioned from one housing system to another, making direct comparisons challenging. Additionally, the authors recorded cortisol levels in horses displaying various stereotypical behaviours. Notably, kicking horses had the highest cortisol levels, while horses that exhibited crib-biting presented the lowest cortisol levels.

#### Negative cortisol–abnormal or stereotypical behaviour relationship

Six of the studies reviewed demonstrate a generally negative relationship between cortisol levels and abnormal or stereotypical behaviour [89–94]. Fejsáková et al. [92] compared the heart rate and cortisol levels of horses exhibiting stereotypical behaviour against those that did not. They found that both heart rate and cortisol levels were lower in horses with stereotypical behaviour, compared to their non-stereotypical counterparts. The study also measured how different kinds of work and horse equipment may influence the heart rate and cortisol levels. The results indicate that more intensive work can lead to increased cortisol levels.

Furthermore, the study found a non-statistically significant but present difference between the horses using bridles and non-bridled horses, with the latter showing lower cortisol levels. Bazzano et al. [89] conducted an examination of cortisol levels in competing versus non-competing horses. The study revealed that among the competing horses, those exhibiting stereotypical behaviour had lower cortisol levels compared to their non-stereotypical counterparts. Additionally, the researchers found that leisure horses exhibited the lowest cortisol levels. In contrast, the findings from Briefer Freymond et al. [90] were opposite to those of McGreevy and Nicol [87], indicating that horses exhibiting stereotypical behaviours generally had higher cortisol levels. Interestingly, cortisol levels increased when stereotypical behaviour was prevented. The researchers measured cortisol in the saliva of horses divided into three groups: non-stereotypical horses, stereotypical horses allowed to engage in their repetitive behaviours, and stereotypical horses that were not allowed to exhibit such behaviour. The experiment included ACTH stimulation, with cortisol levels measured three hours after the injection. The study found that animals exhibiting stereotypies had a higher cortisol response compared to those without stereotypies.

Additionally, stereotypical animals that were prevented from expressing their stereotypical behaviours had a significantly higher cortisol response than those that were allowed to perform the behaviours. This suggests that stereotypical behaviour serves as a coping mechanism. Similar findings were observed in the research conducted by McBride and Cuddeford [93], who compared cortisol levels in stereotypical horses before and after engaging in stereotypical behaviour. The study also measured cortisol levels in stereotypical animals using equipment designed to prevent them from exhibiting these behaviours. Both the crib strap and anti-weave bars resulted in increased cortisol levels in stereotypical horses, with the levels rising significantly for both the anti-weave bar and the crib strap. Additionally, the results indicated that cortisol levels decreased after the abnormal behaviour was exhibited. This suggests a potential coping function for the behaviour. Similar findings were reported by Briefer Freymond et al. [91], where stereotypical horses engaged in crib-biting exhibited significantly lower cortisol levels compared to horses not displaying this behaviour. Cortisol levels and stereotypical behaviours were measured after individuals underwent various tests. Pawluski et al. [94] used two types of materials for a more detailed measurement of cortisol: plasma and faeces. They found that plasma cortisol levels were lower in the evening than in the morning, regardless of whether the individual had worked that day or not. As a behavioural indicator of stress, the study observed ear positioning and withdrawn postures in horses. The study also included haematological data to assess welfare indicators alongside cortisol levels. It was found that individuals with severe vertebral issues had lower evening plasma cortisol levels compared to those with no or slight problems. Similar results were observed in horses with a more backwards ear position. Additionally, horses with anaemia were noted to have lower cortisol levels, suggesting that individuals with poorer welfare generally exhibit lower cortisol levels.

#### No correlation between cortisol level and stereotypical or abnormal behaviour

In four studies, no significant correlation was observed between abnormal or stereotypical behaviours and cortisol levels [95–97, 99]. One study did indicate a slight relationship between these variables; however, it lacked statistical significance [99]. All four studies included locomotor and/or oral stereotypies (highlighted in Supplementary Table 1). Additionally, one study compared eating time in stereotypical and non-stereotypical horses and discovered that the stereotypical horses ate longer than the non-stereotypical ones [95]. Hemmann et al. [97] included measurements of ghrelin, cortisol, adrenocorticotropic hormone and beta-endorphin in stereotypical and non-stereotypical horses. No correlation was found between cortisol levels and stereotypies.

#### Unclear relationship or other factors measured

Three studies examined the correlation between various factors and cortisol levels [100–102]. Freire et al. [100] specifically assessed the effect of diet type (high- and moderate-grain diets) on the occurrence of stereotypical behaviour and cortisol levels. One of the key findings was that horses exhibiting stereotypical behaviours tended to eat more slowly than those without such behaviours. No correlation has been found between cortisol levels and the type of diet. In their study, Pessoa et al. [102] compared working horses based on the amount of time they spent in turnout versus in the stable. They found that horses allowed more time outside had significantly lower cortisol levels compared to those that spent more time confined in the stable, indicating that daily movement and access to space could have a positive effect on stress levels. Patiño et al. [101] also examined the differences between stereotypical and non-stereotypical horses, focusing on cortisol levels, gastric pH, and the presence of gastric ulcers. The study found that stereotypical horses, particularly those that engage in crib-biting, had higher morning cortisol levels, making them more susceptible to severe gastric ulcers compared to non-stereotypical horses. Additionally, Pell and McGreevy [98] found a correlation between cortisol levels and stereotypies only in horses expressing oral stereotypies.

The summary of studies on horses is presented in Table 5.

**Table 5:** Summarisation of studies on horses, including type of abnormal behaviour, type of correlation and the reference number

Type of behaviour/stereotypy	Correlation type	Number of studies (total: 21)	References
*Oral stereotypies* (crib-biting, vocalizing stereotypies, bar-biting, windsucking, wood-chewing, sham-chewing, licking and other)	Positive	7	[82–88]
	Negative	4	[89–91, 93]
	No correlation	4	[95–97, 99]
	Unclear	4	[98, 100–102]
*Locomotory stereotypies* (box walking, weaving, head tossing, head nodding and other)	Positive	3	[82, 83, 88]
	Negative	2	[89, 93]
	No correlation	3	[95, 96, 99]
	Unclear	2	[98, 100]
*Redirected behaviour* (coprophagia, bed eating)	Positive	1	[86]
	Negative	0	–
	No correlation	1	[99]
	Unclear	0	–
Other/not specified	Positive	4	[82, 83, 85, 88]
	Negative	2	[92, 94]
	No correlation	2	[95, 99]
	Unclear	1	[102]

### Cortisol level and abnormal/stereotypical behaviour relationship in dogs

#### Positive cortisol–abnormal or stereotypical behaviour relationship

Out of eight studies conducted on dogs, only two showed a positive correlation between cortisol levels and abnormal behaviour, with a more direct relationship evident in those cases [103, 104]. Both studies implemented enrichment and modifications to the dogs'habitats and daily routines, resulting in decreased cortisol levels. Bicudo Nogueira et al. [103] focused on police dogs to explore the relationship between welfare and environmental conditions. They measured cortisol levels before and after the enrichment sessions and found a significant reduction in cortisol levels following these periods. However, no statistically significant differences were observed between the various types of enrichment. Lefebvre et al. [104] presented a less clear relationship between abnormal behaviour and cortisol levels. This study compared military working dogs under distributed and regular training regimes with those under massed and irregular regimes. In this context, a mass regime refers to a training schedule that concentrates a high volume of work or training into a short period, with minimal recovery time between sessions.

In contrast, a regular regime involves more evenly spaced sessions with consistent scheduling. Results indicated that dogs on the regular regime trotted more and vocalised less than those on the mass regime. Furthermore, the mean cortisol level for dogs on regular regimes was significantly lower compared to the mass regime group. The authors noted that cortisol levels in the regular regime group were higher during the first week of the study but decreased in the following weeks.

#### Negative cortisol–abnormal or stereotypical behaviour relationship

No study has demonstrated a direct negative correlation between cortisol levels and abnormal behaviour in dogs.

#### No correlation between cortisol level and stereotypical or abnormal behaviour

Jongman et al. [105], aside from abnormal and stereotypical behaviour) such as pacing, excessive grooming and jumping) and cortisol levels, included factors such as kennel size and exercise regime in their studies. However, they found no correlation between cortisol levels and behavioural changes. The authors suggest that a larger kennel size and contact with other individuals may improve animals’ welfare.

#### Unclear relationship or other factors measured

Five of the studies show unclear results regarding cortisol levels and behavioural changes [11, 106–109]. Romaniuk et al. [109] measured the cortisol levels of dams after a stressful period, such as postpartum, and found elevated levels between weeks 1 and 8. In their study, Dufour et al. [107], which included shelter dogs, researchers noted no correlation between negative behavioural responses to new stimuli and cortisol levels. However, they discovered that dogs with a lower fear of human contact had lower cortisol levels compared to more fearful dogs, suggesting a potential positive correlation.

Braido Arcuri et al. [11] noted a partially positive correlation between stereotypical behaviour and cortisol levels in Rottweilers, but this correlation was not observed in other breeds, such as Dobermans and German Shepherds.

A possible negative correlation and a suggestion of complexity in the stress response of dogs presenting repetitive behaviour were presented by Denham et al. [106]. Within their research, the cortisol/creatinine ratio was measured in dogs presenting repetitive behaviour, pre- and post-exposure to a stress situation – a veterinary examination. Their results show that dogs presenting repetitive behaviour had a decrease in cortisol level post-exposure to the stressor, which was not observed in other groups of dogs, where cortisol levels increased instead. The researchers concluded that dogs exhibiting repetitive behaviours in elevated excitement situations are motivated differently than those that present repetitive or stereotypical behaviour during periods of minimal stimulation.

Lastly, Haverbeke et al. [108] exposed dogs to various stimuli and found an increase in cortisol levels during repetitive challenges. However, they noted that cortisol levels increased less after the second challenge. Some of the repetitive behaviour diminished during the challenge, while other remained the same (circling). Interestingly, the highly repetitive pacing observed during the control period decreased to less repetitive pacing during both obstacles. At the end of the second challenge, pacing became highly repetitive again. The authors suggested that the observed stereotypical behaviour may not be related to cortisol levels or stress exposure, but rather stems from past experiences when the dogs’ welfare was in poorer conditions.

The summary of studies on dogs is presented in Table 6.

**Table 6:** Summarisation of studies on dogs, including type of abnormal behaviour, type of correlation and the reference number

Type of behaviour/stereotypy	Correlation type	Number of studies (total: 8)	References
*Oral and self-directed stereotypies* (licking, overgrooming, self-biting, paw-chewing)	Positive	1	[104]
	Negative	0	–
	No correlation	1	[105]
	Unclear	0	–
*Locomotor stereotypies* (pacing, circling, jumping, spinning, tail chasing)	Positive	1	[103]
	Negative	0	–
	No correlation	1	[105]
	Unclear	3	[11, 106, 108]
*Object-directed stereotypies* (scratching, manipulation of the environment)	Positive	0	–
	Negative	0	–
	No correlation	0	–
	Unclear	2	[11, 108]
*Other/not specified*	Positive	0	–
	Negative	0	–
	No correlation	0	–
	Unclear	2	[107, 109]

For a detailed overview of the studies reviewed—including information on species, type of abnormal behaviour, direction of correlation, and additional relevant notes—please refer to Supplementary Table 1.

## Discussion

The variability in findings across species and study contexts underscores the multifaceted nature of stereotypic behaviours and their relationship to physiological stress markers, particularly cortisol. These behaviours cannot be universally classified as either stress-induced pathologies or purely adaptive responses to chronic stress. Instead, their function appears highly context-dependent, influenced by a convergence of environmental factors, individual histories, and species-specific behavioural repertoires.

A significant number of reviewed studies support the hypothesis that stereotypies correlate with elevated cortisol levels, suggesting that they may arise in response to persistent stress or suboptimal living conditions. This interpretation aligns with earlier models that conceptualise stereotypic behaviours as indicators of poor welfare. However, an equally compelling body of research suggests that, in some cases, these behaviours are associated with *lower* cortisol levels, implying a potential role in stress mitigation. For example, certain equine studies have demonstrated reductions in cortisol levels following stereotypic episodes, such as crib-biting or weaving, which supports the coping hypothesis [90, 91, 93, 94].

One notable pattern observed is that a higher proportion of studies in horses reported negative correlations between cortisol levels and stereotypy compared to those conducted on zoo mammals. This discrepancy may partially stem from methodological differences. Horses, as domesticated animals, are generally easier to handle, allowing for more precise, temporally relevant blood sampling immediately before and after behavioural observations. In contrast, zoo studies often rely on less frequent sampling, sometimes using faecal or hair cortisol as proxies for longer-term HPA axis activity. While these methods are less invasive, they are also subject to lag times and potentially confounding variables, such as environmental contamination or metabolic differences in hormone breakdown.

Species-specific patterns also emerged. For instance, while great apes and bears in zoos often display stereotypies like pacing or head-tossing, the physiological underpinnings of these behaviours appear to differ across taxa. Some studies show clear links between stereotypies and elevated cortisol in primates [11, 35], while others, particularly in carnivores and ungulates, demonstrate inconsistent or inverse relationships [44, 50, 55, 106]. These findings reinforce the idea that stereotypy is not a homogeneous behavioural category but rather a diverse class of responses with potentially different functions and underlying mechanisms.

Further complicating interpretation is the dynamic nature of cortisol. As a hormone sensitive to diurnal rhythms and external stimuli, cortisol levels can fluctuate due to a range of extraneous variables, including time of day, recent handling, enclosure conditions, social grouping, and feeding routines [9, 10]. This temporal sensitivity makes it difficult to draw definitive conclusions from single-point measurements, particularly when using blood samples, which may reflect acute stress caused by the sampling process itself. Dufour et al. [107] emphasise that the act of restraint and blood collection can elevate cortisol levels independently of any behavioural or environmental stressor, thereby confounding results.

Despite these challenges, a consistent trend across many studies is the presence of elevated cortisol levels in animals exhibiting abnormal or repetitive behaviours. While this supports the use of cortisol as one welfare indicator, the complexity of the relationship necessitates a more integrative approach. Several authors have called for the inclusion of additional hormonal indicators, such as testosterone or oxytocin, as well as behavioural and environmental data, to create more nuanced welfare assessments [18, 25, 64, 73]. In particular, fluctuations in androgens may interact with cortisol dynamics, further influencing behavioural expression and stress susceptibility.

In light of these findings, future research should prioritise multi-dimensional designs that include both longitudinal hormonal monitoring and continuous behavioural observation. Studies should also control for external variables such as diet, housing conditions, and social environment, all of which may significantly influence both cortisol levels and stereotypy expression. Enrichment interventions offer a promising avenue, as some studies have found reductions in both cortisol levels and stereotypic behaviours following increased environmental stimulation [37, 54, 61, 69, 70, 103]. However, the effects of enrichment appear to vary among individuals, indicating that personalised or species-specific welfare strategies may be most effective [19, 30, 51, 74].

Ultimately, while cortisol remains a valuable physiological marker, its interpretation should be contextualised within a broader framework that includes behavioural, environmental, and individual-level data. Only through such integrated approaches can researchers accurately discern whether stereotypic behaviours reflect chronic distress or serve as functional adaptations within constrained environments.

## Conclusions

This review highlights the complex and context-dependent relationship between cortisol levels and stereotypic behaviour in captive and domestic animals. While several studies support a positive correlation—interpreting stereotypies as markers of stress and compromised welfare—others suggest that these behaviours may play a functional role in regulating stress, as evidenced by cases where stereotypy coincides with lower cortisol levels.

Such conflicting findings underscore the limitations of relying solely on cortisol as a biomarker of animal welfare. Methodological inconsistencies across studies, including variations in sample type, timing, and frequency, can significantly impact the results. Moreover, the act of sample collection, particularly for blood, may introduce confounding stress responses that obscure the proper relationship between physiological and behavioural data.

Notably, the reviewed literature suggests that stereotypic behaviours may not be uniformly pathological. Instead, they may represent a spectrum of responses shaped by individual differences, environmental context, and underlying neurobiological mechanisms. This necessitates a shift away from one-dimensional interpretations of stress and towards a more nuanced, integrative model.

Future research should employ multifactorial approaches, combining behavioural observation with longitudinal hormonal analysis and environmental monitoring. Incorporating additional biomarkers, such as other stress-related hormones or immune parameters, may also enhance our understanding of how animals experience and cope with captivity-induced challenges. Such research will be critical in refining welfare assessment tools and improving conditions for animals in managed care.

## Supplementary Information

Additional file 1.

## Data Availability

No datasets were generated or analysed during the current study.

## Abbreviations

HPA: Hypothalamic–Pituitary–Adrenal (axis)
EIA: Enzyme Immunoassay
RIA: Radioimmunoassay
ECL: Electrochemiluminescence
HPLC: High-Performance Liquid Chromatography
COVID-19: Coronavirus Disease 2019
FGM: Faecal Glucocorticoid Metabolites
