# Supplementary material for: Do stereotypies help or harm? Exploring the link between cortisol level and abnormal behaviours in animals: a review
Source: Front Zool. 2025 Aug 13;22:20. doi: 10.1186/s12983-025-00576-0 (PMC12344989; doi:10.1186/s12983-025-00576-0)
Supplement: Supplementary file 1 — Additional file 1. [file 12983_2025_576_MOESM1_ESM.pdf]

CAPTIVE MAMMALS				
**LP**	**REFERENCE**	**SPECIES**	**TYPE OF BEHAVIOUR EXAMINED**	**COMMENTS**
**TYPE OF CORRELATION: POSITIVE**				
1	**[12]**	*Macaca spp.*	Stereotypical behaviours such as bobbing, bouncing, pacing, rocking and other were examined along with affiliative behaviours	The study examined housing conditions as a factor influencing both the expression of stereotypies and cortisol levels.
2	**[15]**	*Gorilla gorilla gorilla*	Abnormal behaviour: anticipatory and affiliative behaviour	The authors measured the effect of positive reinforcement training on oxytocin and cortisol levels, finding that cortisol levels were reduced following the training.
3	**[18]**	*Macaca spp.*	Abnormal behaviour such as self-injury, self-directed behaviour with no wounds and stereotypical behaviour	Close human contact was evaluated as a potential stressor in the study.
4	**[22]**	*Macaca spp.*	Abnormal behaviour: anxiety-related behaviour	Relocation was examined as a potential stressor in the study.
5	**[26]**	*Cebus apella*	Stereotypical behaviour such as pacing and head twirls	The authors found a significant correlation only for head twirls, while no such association was observed for pacing.
6	**[28]**	*Cebus apella*	Abnormal behaviour: intra-group aggression, social interactions and vigilance.	Visual human contact was evaluated as a potential stressor in the study.
7	**[35]**	*Panthera tigris tigris*<br>*Panthera pardus fusca*	Stereotypical behaviour such as pacing, repetitive walk, chewing paws and other.	The study incorporated various internal and external factors, including age, sex, enclosure size, presence of enrichment, and caretaker attitudes.
8	**[37]**	*Ailuropoda melanoleuca*	Stereotypical behaviour such as head-bobbing, pacing, food anticipatory	A negative correlation was found between time spent engaging in stereotypy and enrichment. Additionally, the

				authors examined the relationship with the menstrual cycle and identified a positive correlation.
9	**[38]**	*Ursus maritimus*	Stereotypical behaviour such as pacing	-
10	**[41]**	*Cryptoprocta ferox*	Stereotypical behaviour - pacing	The study included the effect of the mating season and observed a positive correlation with the measured variables in males.
11	**[43]**	*Giraffa camelopardalis* *Acinonyx jubatus*	Stereotypical behaviour: locomotor stereotypies and other types of behaviour.	Human contact was assessed as a stressor, with particular focus on the effects of the absence of visitors during the pandemic period and the subsequent transition phase.
12	**[44]**	*Giraffa camelopardalis*	Stereotypical behaviour such as pacing, licking/gnawing of non-food objects and tounge play.	The study examined the death of a companion as a factor influencing cortisol levels and behavior.
13	**[49]**	*Elephas maximus*	Stereotypical behaviours such as pacing and sleep disruptions	The study examined relocation and the establishment of a new herd as potential stressors.
14	**[50]**	*Elphas* sp.	Stereotypical behaviour such as weaving or stereotyped locomotion.	A positive correlation was observed in a single individual within the study.
15	**[52]**	*Tursiops truncatus*	Abnormal behaviour: behavioural diversity	The authors compared the movements and activities of animals, suggesting that stressed individuals tend to exhibit reduced movement and social interaction.
16	**[53]**	*Lasiorhinus latifrons*	Abnormal behaviour: reduction in activity and avoidance of human contact	Close human contact was evaluated as a potential stressor in the study.
17	**[54]**	*Leptailurus serval* *Leopardus pardalis* *Caracal caracal*	Abnormal behaviour: increase in affiliative behaviour.	The addition of enrichment as a factor was examined, with the correlation predominantly observed in ocelots.

18	**[56]**	*Antelope cervicapra*	Abnormal behaviour: moving, resting, social, reproductive and aggressive behaviour changes were examined.	Close human contact was evaluated as a potential stressor in the study.
19	**[58]**	*Moschus chrysogaster*	Stereotypical such us; eating foreign material, rigid licking, wall jumping and other.	The study accounted for housing type, and muscone levels were measured as part of the analysis.
20	**[61]**	*Felis bengalensis*	Stereotypical behaviour such as pacing and hiding.	The study evaluated living conditions and additional enrichment as factors; however, the correlation was not consistently observed with pacing stereotypy.
21	**[64]**	*Neofelis nebulosa*	Abnormal behaviour: anxiety related behaviour	The study categorized animals into "calm" and "anxious" groups for comparative analysis.
22	**[69]**	*Helarctos malayanus*	Stereotypical behaviour such as pacing, head tossing, swaying and other.	The study also compared semi-captive animals with fully captive individuals and included environmental enrichment as a variable in the analysis.
23	**[70]**	*Melursus ursinus*	Stereotypical behaviour such as pacing.	The effect of environmental enrichment was systematically measured in the study.
24	**[72]**	*Callithrix jacchus*	Auto-grooming and scent marking; the presence of contact and alarming calls	The authors included environmental factors by examining differences between outdoor and indoor ambient conditions. The study also considered external variables such as weather conditions and the presence of loud noises from a nearby construction site.
25	**[73]**	*Saguinus oedipus*	Abnormal behaviour: affiliative, aggressive, scent-marking and other behaviours were examined	The study included reproductive hormone levels in the analysis and identified a positive correlation with aggressive behavior.
26	**[75]**	*Papio papio*	Stereotypical behaviour such as pacing, rocking and rhythmic jumping.	The study incorporated enrichment through the use of a computerized learning system.
27	**[76]**	*Pan paniscus*	Self-directed - hair-plucking	The study observed a significant correlation exclusively in female subjects, with no corresponding association detected in males.

28	**[79]**	*Rhinopithecus roxellana*	Stereotypical and aggressive behaviour	The study measured group division and relocation as factors influencing stress levels.
29	**[81]**	*Moschus chrysogaster*	Stereotypical behaviour such us eating foreign material, rigid licking, wall jumping and other.	The study compared high-density and low-density groups and additionally measured musk secretion levels.
**TYPE OF CORRELATION: NEGATIVE**				
1	**[17]**	*Gorilla gorilla gorilla*<br>*Pan troglodytes*	Abnormal behaviour such as coprophagy, faecal manipulation and other were examined. The affiliative behaviours	The study included environmental factors in the examination, alongside the presence of enrichment.
2	**[19]**	*Macaca spp.*	Abnormal and stereotypical behaviour (pacing, bitting the mesh)	The study included various types of enrichment as part of the experimental design.
3	**[27]**	*Macaca spp.*	Stereotypical behaviour such as whole-body stereotypies, self-directed stereotypies, fine motor stereotypies and other	-
4	**[33]**	*Acinonyx jubatus*	Abnormal behaviour: behavioural diversity	The authors suggest that elevated stress levels may lead to a reduction in behavioral diversity.
5	**[46]**	*Elephas maximus*	Stereotypical behaviour – not specified	The authors also assessed body condition scores, foot health scores, and wound scores in the animals.
6	**[48]**	*Loxodonta africana*	Stereotypical behaviour - swaying	-
7	**[63]**	*Panthera pardus*<br>*Uncia uncia*<br>*Leptailurus serval*	Stereotypical behaviour such as pacing	The study included external factors, such as the presence of a construction site, as potential stressors.
8	**[65]**	*Ursus maritimus*<br>*Neofelis nebulosa*	Stereotypical behaviour	-

9	**[71]**	*Callithrix jacchus*	Stereotypical behaviour such as scratching, autogrooming, presence of anhedonia and anxiety behaviours	The study also investigated juvenile depression in primates and evaluated various treatment methods.
**TYPE OF CORRELATION: NO CORRELATION**				
1	**[13]**	*Ailuropoda melanoleuca*	Abnormal behaviour: changes in mating behaviour and success	The study also considered additional hormones and physiological parameters in the analysis.
2	**[14]**	*Pongo pygmaeus*	Stereotypical and stress related behaviours such as manual manipulation, self-directed, cage biting and observer-directed	The study utilized computerized testing to assess behavioral responses following exposure to a stressor; however, no significant differences were observed in cortisol levels.
3	**[16]**	*Pan troglodytes*	Abnormal behaviour: aggressive behaviours	The study included environmental factors and relocation as variables influencing the outcomes.
4	**[20]**	*Macaca spp.*	Stereotypical behaviour such as pacing, self-biting, self-clasping and other	The study also measured the effect of enrichment on the subjects.
5	**[21]**	*Macaca spp.*	Abnormal behaviour with rating 1-15 proposed by the authors. Included behaviour such as self-injuring, locomotor stereotypies, overgrooming and other.	The study measured products of proopiomelanocortin as part of the hormonal analysis.
6	**[24]**	*Macaca spp.*	Abnormal and stereotypical behaviour such as self-clasping	The study included only male individuals and investigated factors such as housing system, presence of diarrhea, and body weight.
7	**[29]**	*Macaca spp.*	Stereotypical behaviour: Mmostly self-directed including freezing with saluting and digit sucking	The study compared primates rejected by their mothers with those that were maternally reared. The methodology also included examination of cerebrospinal fluid, measurement of oxytocin and corticotropin-releasing hormone, as well as multimodal neuroimaging.
8	**[30]**	*Acinonyx jubatus*	Stereotypical behaviour such as self-mutilation, head-rolling and pacing	The study included factors such as environmental changes, sex, age, housing conditions, and other relevant variables.

9	**[31]**	Panthera leo<br>*Panthera tigris*	Stereotypical behaviour such as passing, pacing and other types of behaviour.	The presence of an external factor—the construction site—was examined as a potential stressor. Cortisol levels increased during the construction period; however, no correlation was found with behavioral changes.
10	**[34]**	*Panthera tigris*	Abnormal and stereotypical behaviour such as pacing; also, resting and other types of behaviour	The study compared captive animals with those living in semi-natural habitats and examined different forms of captivity. Behavioral differences were observed between groups; however, differences in cortisol levels were not statistically significant.
11	**[39]**	*Ailuropoda melanoleuca*	Abnormal and stereotypical behaviour such as pacing or self-sucking	The study examined the effect of transport, finding that cortisol levels increased during transport, although no corresponding behavioral changes were observed.
12	**[40]**	*Ailuropoda melanoleuca*	Stereotypical behaviour such as turning around, biting the rail, "step by step" behaviour.	The study investigated various types of social housing in the animals and included gut microbiota analysis alongside other physiological and behavioral parameters.
13	**[51]**	*Loxodonta africana*	Stereotypical behaviour – swaying.	The study revealed that scheduled activities in elephants may reduce the occurrence of stereotypical behavior. Additionally, cortisol levels varied among individual animals.
14	**[62]**	*Leptailurus serval*	Stereotypical behaviour – pacing.	The study explored the effect of encounter programs on adrenocortical activity. The researchers observed a decrease in the duration of stereotypical behaviors; however, no correlation with cortisol levels was detected.
15	**[66]**	*Panthera onca*	Stereotypical behaviour including pacing, over-grooming, tail-sucking and other.	The study included a larger population of captive jaguars in North America.
16	**[68]**	*Uncia uncia*	Stereotypical behaviour – pacing.	The study examined the effect of an active foraging device, with the authors highlighting the complexity of behavior and the influence of additional external factors.

17	**[77]**	*Hapalemur alaotrensis*	Abnormal and stereotypical behaviour such as pacing or self-scratching.	The study examined the effect of olfactory enrichment, assessing sexual activity and hormone levels. No significant changes were observed in cortisol levels.
18	**[80]**	*Aotus spp.*	Stereotypical behaviour such as pacing and somersaults.	The study examined juvenile individuals subjected to early parental deprivation and found a positive correlation between deprivation status and infection rate.
**TYPE OF CORRELATION: UNCLEAR**				
1	**[23]**	*Macaca spp.*	Abnormal and stereotypical behaviour; including sitting together, locomotion behaviour	The study found that maternal separation resulted in lasting alterations in cortisol levels and behavior in rhesus monkeys. However, a direct correlation between cortisol levels and stereotypical behaviors was not explicitly reported.
2	**[25]**	*Macaca spp.*	Abnormal and stereotypical behaviour including aggression, fecal fainting and stereotypical behaviour.	The researchers observed reduced cortisol, follicle-stimulating hormone, and estradiol levels, along with anovulatory bleeding potentially linked to stress. However, the study did not specifically address stereotypical behaviors or their correlation with cortisol. Notably, menstruating individuals appeared to experience lower stress levels than non-menstruating counterparts.
3	**[32]**	*Panthera tigris*	Not specified.	The study compared captive, re-wilded, and free-ranging tigers, finding no significant differences in cortisol levels between these populations.
4	**[36]**	*Ailuropoda melanoleuca*	Abnormal and stereotypical behaviour such as pacing, head tossing. The collar-directed behaviour was examined separately.	The study examined the effect of radiocollaring on the animals and found no significant changes in stereotypic behaviors, activity levels, or cortisol levels in any of the four pandas following the fitting of radiocollars. These results suggest that radiocollaring did not induce stress-related behavioral or physiological alterations in the subjects.
5	**[42]**	*Camelus dromedarius*	Stereotypical and abnormal behaviour such as bar mouthing or head shaking.	The study observed that certain behaviors, including inactivity and stereotypies, exhibited daily rhythmicity with peaks at specific times of the day. This finding suggests that hormonal fluctuations, such as those in cortisol levels, may influence the expression of these behaviors.

6	**[45]**	*Camelus dromedarius*	Abnormal and stereotypical behaviour - negative interactions	The study examined the external factor of performing rides for guests at the zoo. The authors suggest that the use of animals for entertainment rides does not constitute a significant stressor.
7	**[47]**	*Loxodonta africana*	Abnormal and stereotypical behaviour such as weaving or head bobbing	The researchers investigated the effects of aversive geofencing devices on captive Asian elephants, focusing on behavioral and physiological stress indicators. The study found no lasting stress effects during the short-term period. Although the authors emphasize the need for long-term studies, a tentative positive correlation was observed.
8	**[55]**	*Pongo pygmaeus*<br>*Symphalangus syndactylus*<br>*Hylobates lar*<br>*Varecia rubra*<br>*Alouatta caraya*	Abnormal and stereotypical behaviour such as pacing and self-scratching	The authors identified both positive and negative correlations with cortisol levels, depending on the group. Scent enrichment was evaluated as a factor potentially influencing stress responses.
9	**[57]**	*Axis porcinus*	Abnormal behaviour: behavioural changes	The study examined seasonal changes and other environmental conditions in conjunction with enclosure size.
10	**[59]**	*Moschus berezovskii*	Stereotypical behaviour such as walking back and forth and constant jumping	The study does not provide direct evidence but suggests that crowding may influence stress levels, potentially affecting behaviors in captive forest musk deer.
11	**[60]**	*Canis rufus*	Not specified.	The study investigates the relationship between dietary types and fecal glucocorticoid metabolite (FGM) concentrations, a biomarker of stress, in zoo-managed red wolves. The authors found that individuals fed a commercial kibble diet exhibited both higher baseline and elevated FGM concentrations over time compared to those receiving a mixed diet of commercial kibble and meat. These findings suggest that diet composition can significantly influence stress levels in captive red wolves.
12	**[67]**	*Panthera pardus*	Not specified.	The study examined the effects of various types of enrichment on the subjects.

13	**[74]**	*Sapajus libidinosus*	Abnormal (behaviour potentially indicative of stress) and stereotypical behaviour such as pacing, head twirls, bouncing self-scratching and other.	The findings indicate that individual differences in coping styles influence physiological stress responses in capuchins. However, the authors suggest that the observed decrease in cortisol levels among animals exhibiting stress-related behaviors may reflect a calming effect of certain behaviors, resulting in a negative correlation with cortisol. Conversely, for other types of behaviors, a positive correlation with cortisol levels was noted.
14	**[78]**	*Lemur catta*	Abnormal and stereotypical behaviour - diversity of beahviour	The study evaluated close human contact as a potential stressor. While no correlation was found between human contact and stereotypic behaviors, cortisol levels were observed to be lower during off-peak hours when fewer tourists were present. Additionally, fecal cortisol metabolite levels decreased in periods with reduced tourist presence.

HORSES

LP	REFERENCE	TYPE OF ABNORMAL BEHAVIOUR	COMMENTS
**TYPE OF CORRELATION: POSITIVE**			
1	**[82]**	Abnormal behaviours such as oral stereotypies, locomotor stereotypies, social problems and training behaviours.	The authors investigated various types of diets and other related parameters. They also compared cortisol levels with dehydroepiandrosterone (DHEA) levels.
2	**[83]**	Stereotypical behaviour such as kicking, biting, weaving and crib-biting	The study also examined different housing systems. A reduction in stereotypic behavior was correlated with a decrease in cortisol levels.
3	**[84]**	Stereotypical behaviour – crib-biting	The authors suggested potential habituation to stressors, as cortisol levels did not increase in stereotypic horses following exposure to the stressor. Overall, cortisol levels were higher in stereotypic individuals.
4	**[85]**	Stereotypical behaviour such as excessive licking, box-kicking, crib-biting	The study included housing as a potential stress factor influencing behavioral and physiological responses.
5	**[86]**	Oral stereotypical behaviour such as sham chewing and redirected behaviour like coprophagy.	The authors analyzed different types of diets and suggested that feeding adaptations may reduce the occurrence of abnormal behavior.

6	**[87]**	Stereotypical behaviour – crib-biting	Cortisol levels did not increase when crib-biting behavior was prevented.
7	**[88]**	Stereotypical behaviours such as box walking, weaving, bar-biting and more.	The study included an analysis of white blood cells. The authors suggested that single housing may act as a stressor for the animals.
**TYPE OF CORRELATION: NEGATIVE**			
1	**[89]**	Stereotypical behaviour such as crib-biting, weaving and box walking	The authors compared competing and non-competing horses. Horses exhibiting stereotypic behaviors had lower cortisol levels.
2	**[90]**	Stereotypical behaviour such as crib-biting	The authors aimed to prevent stereotypic behavior in affected animals and observed an increase in cortisol levels.
3	**[91]**	Stereotypical behaviour – crib-biting	The authors suggested that stereotypic behavior may serve an adaptive function.
4	**[92]**	Stereotypical behaviour – not specified	The authors suggested that stereotypies have a habitual or self-reinforcing nature. Heart rate and 11,17-dioxoandrostanes were also compared with cortisol levels and behavioral data. Behavioral data were collected through questionnaires completed by caretakers, owners, or riders.
5	**[93]**	Stereotypical behaviours such as crib-biting and weaving	The authors aimed to prevent stereotypic behavior in affected animals and observed an increase in cortisol levels.
6	**[94]**	Abnormal behavioru – abnormal ear position	The authors suggested that chronic stress may lead to a compromised stress response in animals. The study included an analysis of blood cells.
**TYPE OF CORRELATION: NO CORRELATION**			
1	**[95]**	Stereotypical behaviours such as cribbing and weaving along with other types of behaviour.	The study did not find significant differences in plasma cortisol levels between stereotypic and non-stereotypic horses, suggesting that cortisol concentrations may not be directly correlated with the expression of these stereotypies. Additionally, stereotypic horses were observed to eat for longer durations than non-stereotypic horses.

2	**[96]**	Stereotypical behaviour - oral and motor stereotypies.	No correlation was found between cortisol levels and either locomotor or oral stereotypies.
3	**[97]**	Stereotypical behaviour – crib-biting.	The study measured ghrelin, cortisol, adrenocorticotropic hormone, and beta-endorphin levels in stereotypic and non-stereotypic horses. No correlation was found between cortisol levels and stereotypic behaviors.
4	**[99]**	Stereotypical behaviour (aerophagia, weaving etc.) and other abnormal behaviour (coprophagia, bed eating etc.)	The study was conducted on police horses. The observed correlation was not statistically significant.
**TYPE OF CORRELATION: UNCLEAR**			
1	**[98]**	Stereotypical behaviour - oral (crib-biting, windsucking) and locomotory stereotypies (weaving, box walking)	The authors also measured beta-endorphin levels in the horses studied. They found no significant differences in mean plasma or salivary cortisol concentrations between horses exhibiting stereotypic behaviors and those that did not. A correlation was observed only in horses with oral stereotypy.
2	**[100]**	Stereotypical behaviour such as crib-biting and weaving	The study also measured feeding duration in horses. The authors found that stereotypic horses tended to eat more slowly than non-stereotypic horses. No correlations were found with the type of diet.
3	**[101]**	Stereotypical behaviour such as crib-biting.	The study examined gastric pH and the presence of gastric ulcers alongside cortisol levels. The authors found that the presence of ulcers may be correlated with the occurrence of crib-biting behavior.
4	**[102]**	Abnormal behaviour such as standing alert, drowsy, vocalizing stereotypies, fearful and other were evaluated	The study on working police horses aimed to assess how confinement influences stress and the occurrence of undesirable behaviors. Cortisol levels were significantly higher in horses that spent 24 hours confined to stalls. Some stress-indicative behaviors were also more frequent in horses spending extended time in stalls. These results suggest a positive correlation between confinement and stress-related responses.

DOGS			
**LP**	**REFERENCE**	**TYPE OF ABNORMAL BEHAVIOUR**	**COMMENTS**
**TYPE OF CORRELATION: POSITIVE**			
1	**[104]**	Different types of behaviour including stereotypical behaviour such as: repetitive trot and walk (categorized as stereotypy)	The authors investigated the impact of a regular training regimen as a form of environmental enrichment for dogs.
2	**[103]**	Different types of behaviour including stereotypical behaviour such as circling, pacing, head twirls etc.	The study, conducted on police dogs, evaluated the effects of enrichment sessions on stress levels.
**TYPE OF CORRELATION: NO CORRELATION**			
1	**[105]**	Different types of stereotypical or abnormal behaviour including pacing, licking, excessive grooming and jumping.	The authors examined factors such as kennel size and exercise regimen. They suggested that larger kennel dimensions and social contact with conspecifics may contribute to improved welfare in dogs.
**TYPE OF CORRELATION: UNCLEAR**			
1	**[106]**	Stereotypical behaviour such as bouncing, circling, pacing and spinning	The authors suggested a possible negative correlation. The cortisol-to-creatinine ratio was measured, and dogs exhibiting repetitive behaviors showed lower post-stress cortisol levels compared to other groups.
2	**[108]**	Different types of behaviour including stereotypies such as pacing and manipulation of the environment.	The authors exposed dogs to various stimuli and observed that some stereotypic behaviors diminished in response, while others persisted.
3	**[107]**	Dogs behaviour assessment in three different situations and environmental conditions.	The study on shelter dogs found no significant correlation between behavioral stress responses and cortisol levels.
4	**[109]**	A three-step test for behaviour assessment in dogs – the behavioural response to stranger approaching.	The study included the postpartum period as a stressor in dams. Cortisol levels were elevated during weeks 1 to 8 postpartum, accompanied by observable changes in affiliative behavior during the initial weeks.
5	**[11]**	Different types of behaviour including moving, resting and stereotypical behaviour such as circling,	A positive correlation between stereotypic behavior and cortisol levels was identified exclusively in one specific breed.

		manipulation of the environment and repetitive walking	
